# Clinical Insights in RNA-Binding Protein Motif 20 Cardiomyopathy: A Systematic Review

**DOI:** 10.3390/biom14060702

**Published:** 2024-06-14

**Authors:** Marika Martini, Maria Bueno Marinas, Ilaria Rigato, Kalliopi Pilichou, Barbara Bauce

**Affiliations:** 1 Department of Cardiac, Thoracic, Vascular Sciences and Public Health, University of Padova, 35128 Padova, Italy; marika.martini.1@phd.unipd.it (M.M.); maria.buenomarinas@unipd.it (M.B.M.); barbara.bauce@unipd.it (B.B.); 2 Azienda Ospedaliera di Padova, 35128 Padova, Italy; ilaria.rigato@unipd.it

**Keywords:** RNA-binding protein motif 20 (*RBM20*), cardiomyopathy, phenotypic expression

## Abstract

Dilated cardiomyopathy (DCM) is a common cause of heart failure (HF) and heart transplantation (HTx), with genetic factors playing a significant role. In recent years, the RNA-binding protein motif 20 (*RBM20*), which affects the gene splicing of various proteins with different cellular functions, was identified as the first DCM gene with regulatory properties. Variants of *RBM20* have been associated with severe forms of DCM. The aim of this critical systematic review was to analyse *RBM20* cardiomyopathy clinical features and outcomes. According to PRISMA guidelines, a search was run in the PubMed, Scopus and Web of Science electronic databases using the following keywords: “*RBM20*”; “cardiomyopathy”; “arrhythmias”; “heart failure”. A total of 181 records were screened, of which 27 studies were potentially relevant to the topic. Through the application of inclusion and exclusion criteria, eight papers reporting 398 patients with *RBM20* pathogenic variants were analysed. The mean age at presentation was 41 years. Familiarity with cardiomyopathy was available in 59% of cases, with 55% of probands reporting a positive family history. Imaging data indicated a mild reduction of left ventricular ejection fraction (mean LVEF 40%), while tissue characterization was reported in 24.3% of cases, showing late gadolinium enhancement in 33% of patients. Composite outcomes of sustained monomorphic ventricular tachycardia or ventricular fibrillation occurred in 19.4% of patients, with 12% undergoing HTx. There were no gender differences in arrhythmic outcomes, while 96.4% of patients who underwent HTx were male. In conclusion, *RBM20* cardiomyopathy exhibits a severe phenotypic expression, both in terms of arrhythmic burden and HF progression.

## 1. Introduction

Dilated cardiomyopathy (DCM) is a common heart muscle disease and one of the leading causes of end-stage heart failure (HF) and heart transplantation (HTx) [1]. The aetiology of DCM exhibits significant heterogeneity, with the familial subtype accounting for 30% to 50% of cases [2,3]. In contrast to Hypertrophic Cardiomyopathy (HCM) and Arrhythmogenic Cardiomyopathy (ACM), where a few genes account for the majority of cases, dilated cardiomyopathy (DCM) is associated with variants in a wide range of genes with diverse cellular functions [4]. In the last few years, the gene for RNA-binding protein motif 20 (*RBM20*) was identified as the first DCM gene with regulatory properties.

It impacts the posttranscriptional splicing of numerous genes, extending its regulatory influence to the orchestration of complex gene expression patterns, unlike common DCM genes that have only structural or metabolic properties [5]. Since Titin (*TTN*) was one of the first genes shown to be regulated by *RBM20*, much of the research attention in *RBM20* cardiomyopathy has focused on *TTN*. Further studies have shown that *RBM20* regulates calcium handling, suggesting that *TTN* missplicing is just one of the implicated pathogenic pathways [6]. However, the phenotypic expression of *RBM20* cardiomyopathy was observed to be more severe than that in *TTN* variant carriers, exhibiting a greater propensity for arrhythmic events and progression to HF [3]. The aim of this critical systematic review of the literature was to assess the prevalence, clinical, and instrumental features of *RBM20* cardiomyopathy, as well as its outcomes.

## 2. Methods

### 2.1. Protocol Registration

The systematic review protocol of this study was registered on PROSPERO, International prospective register of systematic reviews (Center for Reviews and Dissemination, University of York, York, UK) (registry number CRD42024506023).

### 2.2. Study Plan

This study was conducted according to PRISMA guidelines (http://www.prismastatement.org/) (accessed on 6 February 2024). A search was run in the PubMed, Scopus and Web of Science electronic databases for clinical studies that investigated RBM20 patients. We collected published research using the following search items: “RBM20” AND (“cardiomyopathy” OR “arrhythmias” OR “heart failure”). MeSH terms and keywords were combined accordingly on the aforementioned databases. The reference list of all the included articles was accurately screened in order to identify other pertinent studies. The “Related Articles” option on the PubMed homepage was also considered. No restriction on publication date was applied. Titles and abstracts of articles available in the English language were also evaluated. The full texts of the publications identified were screened for original data, and the references in the articles retrieved were checked manually for other relevant studies. The literature search has been updated to 6 February 2024.

### 2.3. Inclusion and Exclusion Criteria

Studies were included when the following general criteria were met: (1) articles were original reports; (2) reports were published in the English language. In articles investigating *RBM20* genetic pathways by in vitro or animal models, only the clinical aspects concerning patients with the mutation were analysed, in line with the review’s clinical purpose. Editorials, reviews and case reports were excluded.

### 2.4. Data Extraction

**Figure 1:**
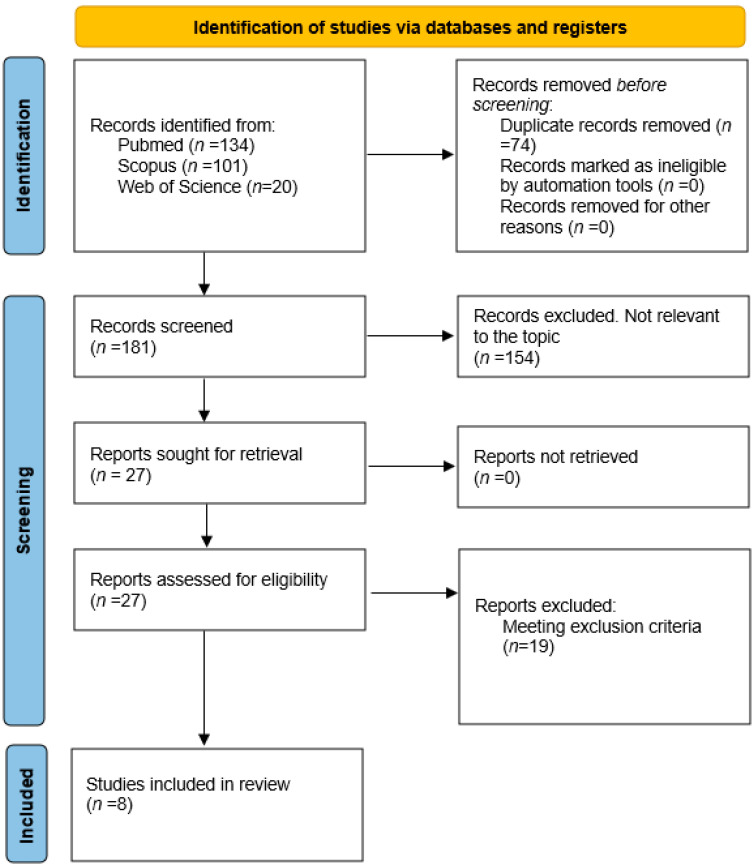
PRISMA flow diagram summarizing the literature review and inclusion/exclusion process.

Two of the authors (M.M. and B.B.) extracted the data from the selected articles. Disagreements were dealt with by discussion among the team members. Details of the search process and study selection are shown in Figure 1. Information extracted from the studies included the title, name of the first author, year of publication, country of study population and qualitative description of target population. Each included study was analysed to extract all available data and ensure the eligibility of every single patient. For our review, we considered the following patients’ data: presence of genetic variant and its prevalence in DCM cohort, onset of the diseases, family history of sudden cardiac death (SCD), family history of cardiomyopathy, disease penetrance, phenotypic expression, sex difference in phenotypic expression, onset of disease, ECG features, echocardiographic features in terms of left ventricle (LV) dilation and dysfunction, cardiac magnetic resonance features in terms of LV dilation, dysfunction and late gadolinium enhancement (LGE) presence and pattern, ICD implantation, major arrhythmic events (sustained ventricular tachycardia (SVT), ventricular fibrillation (VF)), HF occurrence, end-staged HF and HTx.

The statistical data were analysed with version 26 of SPSS (SPSS Inc., Chicago, IL, USA).

## 3. Results

The studies included in the systematic review are summarized in Table 1, while Table 2 specifies the genetic variant reported in each study.

### 3.1. Study Retrieval

A total of 255 titles were retrieved (134 from PubMed, 101 from Scopus and 20 from Web of Science). After removing duplicates, 181 unique titles were screened, resulting in the identification of 27 potentially relevant studies. Full-text screening of these articles led to the exclusion of 19 studies due to non-compliance with the inclusion/exclusion criteria. Overall, eight papers were finally considered for this review (Table 1) [5,7,8,9,10,11,12,13]. A PRISMA flow diagram illustrates the information through the different phases of the literature review (Figure 1).

### 3.2. Clinical Features of Patients with RBM20 Cardiomyopathy

Overall, eight studies involving 398 patients with an *RBM20* pathogenetic variant were analysed. Two studies reported a prevalence of *RBM20* variants of approximately 3% in DCM cohorts [5,7]. Data on the status of the probands were available for 329 cases (82%), with 109 (33%) identified as probands and 220 (66%) as family members. In 258 cases (64%), gender information was reported, demonstrating an equal distribution between both sexes (male *n* = 129, 50%). Among the 83 probands, medical history data regarding a family history of cardiomyopathy were available (*n* = 58, 70%), with 44 (55%) of 80 probands showing positive familiarity for SCD. The mean age at presentation was 41 years.

In two studies out of a total of 210 patients (52%), the average age of onset was reported for males (28.5 years) and females (44.8 years) [9,12].

Among 204 patients (51%), symptomatic presentation at the initial assessment was reported, with 102 (50%) of patients exhibiting symptoms. Dyspnoea was present in 74 patients (36%) at first evaluation. Data on ICD implantation were available for 313 patients (78.6%), with 124 patients (40%) receiving an ICD for both primary and secondary prevention.

### 3.3. Electrocardiographic Features of Patients with RBM20 Cardiomyopathy

Baseline ECG data were available for 194 (48%) patients, revealing a normal mean heart rate (HR) of 69.5 bpm, a mean PR interval of 150 ms and a mean QRS duration of 101 ms. Out of 164 patients (41%), 10 (6%) presented with left bundle branch block (LBBB). No other electrocardiographic features were reported.

### 3.4. Imaging Features of Patients with RBM20 Cardiomyopathy

Data on LV ejection fraction (LVEF) were available for 299 patients (75%), indicating a mean LVEF of 40%, while mean LV dimensions were within normal ranges (LV end-diastolic diameter, LVEDD) of 60 mm. An analysis of right ventricular (RV) parameters was available in only 146 patients, reporting RV dilation in 17 cases (11%).

**Figure 2:**
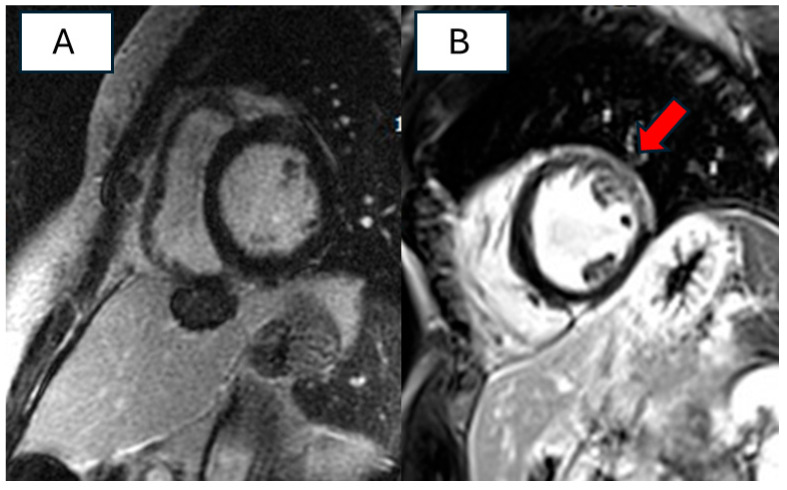
Contrast-enhanced short-axis images of two probands with RBM20 pathogenetic mutation and high arrhythmic burden. (**A**) Absence of LGE (**B**) Non-ischemic LGE in the anterior and antero-later walls.

Regarding cardiac magnetic resonance imaging (CMR), data on morpho-functional characteristics were available for 44 patients (11%), revealing a mild reduction in LVEF (mean 45%) and LV dilation (120.5 mL/mq). Tissue characterization was reported in 97 patients (24%), showing the presence of late gadolinium enhancement (LGE) in 32 patients (33%). Figure 2 shows differences in tissue characterization between two probands patients with *RBM20* cardiomyopathy and high arrhythmic burden.

### 3.5. Outcome Data of Patients with RBM20 Cardiomyopathy

The outcome data were reported varied across the studies. Out of 232 patients, 32 (14%) developed atrial fibrillation (AF); appropriate ICD intervention was reported in 36 patients out of 111 (32%); the composite outcomes of sustained monomorphic ventricular tachycardia or ventricular fibrillation were present in 39 patients out of 201 (19%); SCD was reported in 6 out of 182 cases (3%); finally, 38 out of 317 patients (12%) underwent HTx.

### 3.6. Sex-Related Disease Expression in RBM20 Cardiomyopathy

Two studies have investigated the phenotypic presentation of the disease based on sex [9,12]. Proband status was similar in the two groups (male *n* = 22, female *n* = 25). The mean age at diagnosis was lower for men (28.5 vs. 45 years). Moreover, men presented with a lower left ventricular ejection fraction (mean 37% vs. 45%). Among 74 patients receiving an ICD for both primary and secondary prevention, no differences were observed between the two sexes (male *n* = 36 (49%) vs. female *n* = 38 (51%).

In terms of the composite arrhythmic outcome encompassing SCD, VF and SVT, out of 39 patients, 20 were male (51%) and 19 were female (49%), indicating no significant differences between the genders. On the contrary, of 28 patients across the two studies who underwent cardiac transplantation, 27 were male (96%).

## 4. Discussion

**Figure 3:**
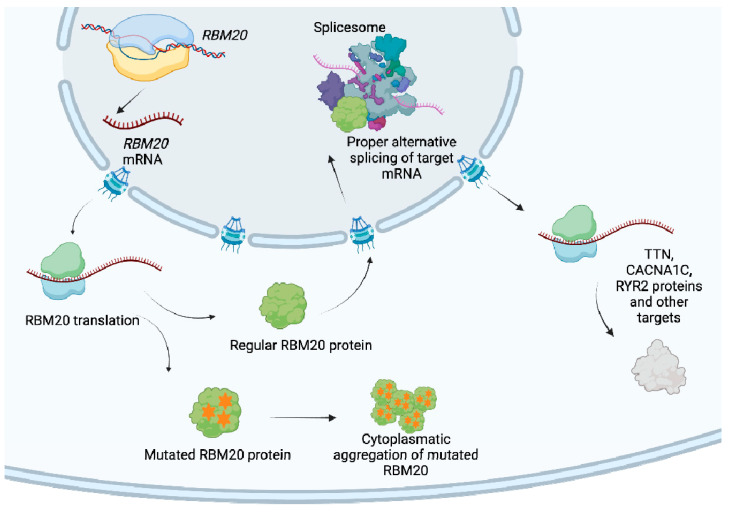
Schematic representation of *RBM20* synthesis, transportation and regular/pathological function. *RBM20* mRNA is synthesized in the nucleus and then exported to the cytoplasm, where it is translated into protein. Regular *RBM20* returns to the nucleus, binds the spliceosome and regulates alternative splicing of its targets (i.e., TTN, RYR2). In case of genetic variants, mutated *RBM20* protein is not able to translocate into the nucleus, and, as a consequence, alternative splicing is not feasible. Created with BioRender.com.

*RBM20* cardiomyopathy is a genetically heterogeneous condition associated with variants in the *RBM20* gene, which plays a role in the regulation of cardiac gene expression and function. Figure 3 comprehensively summarizes the main genetic pathways associated with the *RBM20* gene. This study provides a comprehensive analysis of the clinical, electrocardiographic, imaging and outcome data of patients with *RBM20* cardiomyopathy, shedding light on the varied phenotypic expressions and sex-related differences in disease manifestation.

### 4.1. Genetic Background

Most of the initially identified genes associated with DCM encode proteins crucial for the cytoskeleton or contractile machinery of cardiac myocytes [16]. These proteins play a direct role in generating and transmitting contractile force through interactions with other cellular components. However, a deeper understanding of DCM pathobiology has emerged with the discovery of genetic variants that disrupt myocardial function through alternative mechanisms. For instance, *LMNA*, whose pathogenetic mutations are implicated in an aggressive form of cardiomyopathy along with non-cardiac manifestations, encodes a nuclear membrane protein expressed ubiquitously [17].

In recent years, the *RBM20* gene has been identified as the first DCM gene with regulatory properties, influencing the posttranslational splicing of numerous sarcomeric and calcium-handling genes within cardiomyocytes [18]. Over thirty splicing targets of *RBM20* have been reliably identified to date [8,19]. From a pathogenetic perspective, some key targets include Titin (*TTN*), one of the proteins crucial for myocardial contraction, Ryanodine Receptor 2 (*RYR2*) [20], which encodes for a calcium-ion channel essential for calcium homeostasis and Ca2+/calmodulin-dependent (CaMKII- protein kinase II), also involved in calcium-handling [21]. An aggregation of mutated *RBM20* granules within the cytoplasm can also occur due to a possible disruption of the protein nuclear transport process, especially when localized in the RS domain [22]. Not every genetic variant could lead to cytoplasmatic aggregation, highlighting the clinical heterogeneity of the disease and the need to further investigate the different effects of each genetic variant [23,24,25].

### 4.2. Clinical Features of Patients with RBM20 Cardiomyopathy

The analysis of eight studies, encompassing 398 patients with *RBM20* pathogenetic mutations, reveals significant insights into the epidemiology and clinical characteristics of DCM associated with *RBM20* mutations. Notably, the prevalence of *RBM20* mutations within DCM cohorts ranged around 3%, indicating its importance as a genetic contributor to this condition.

Furthermore, a notable proportion of probands demonstrated a positive family history of cardiomyopathy, corroborating the high familiar penetrance reported in several studies [9,11]. A detailed analysis of the literature reveals a lack of clinical studies performing sub-analyses on family members to gain deeper insights into penetrance and phenotypic expression. Pantou et al. described a family of six individuals carrying an *RBM20* gene variant, three of whom met the diagnostic criteria for DCM. All three patients exhibited episodes of sustained VT, and one had a conduction defect on a 12-lead ECG. Additionally, one patient showed echocardiographic signs of early DCM, demonstrating that this gene variant is characterized by variable expression within family members [26].

Additionally, a significant number of cases had a family history of SCD, further emphasizing the serious clinical consequences of *RBM20* genetic variants and the necessity of familial screening.

In DCM, the age of clinical onset exhibits significant variability, ranging from 20 to 60 years [27]. In our study, the mean age at presentation was found to be in the early forties, with notable variations observed in the age of onset between genders, suggesting potential gender-specific differences in disease manifestation. Symptomatic presentation was prevalent, with dyspnea being a common initial symptom, underscoring the impact of *RBM20* mutations on cardiac function.

### 4.3. Instrumental Features of RBM20 Cardiomyopathy

Electrocardiographic findings demonstrated relatively normal baseline parameters, albeit with occasional instances of left bundle branch block (LBBB). To date, no study has identified specific electrocardiographic characteristics associated with *RBM20* cardiomyopathy or a higher rate of conduction disturbance, unlike the findings observed in *LMNA* cardiomyopathy [28].

Different imaging studies revealed LV dysfunction, with a significant proportion of patients demonstrating mildly reduced EF. Additionally, cardiac magnetic resonance imaging (CMR) provided valuable insights into morpho-functional characteristics, with the significant limitation that less than half of the patients across the various studies had undergone the examination.

The available data confirm the presence of dilation and mild left ventricular dysfunction, as evidenced by echocardiography. Interestingly, more than two-thirds of the patients did not exhibit late gadolinium enhancement at tissue characterization, suggesting that LV dysfunction and the presence of fibrosis did not share the same pathogenetic pathway in DCM [29].

### 4.4. Outcomes Data and Gender Difference in Patients with RBM20 Cardiomyopathy

The outcomes data underscored the arrhythmic nature of *RBM20* cardiomyopathy, with notable occurrences of atrial fibrillation (AF), sustained ventricular tachyarrhythmias, and SCD. The studies included in the review are unanimous in describing a high arrhythmic burden with no gender difference.

Interestingly, Cannie et al. described the occurrence of MVA even in the presence of a mild reduction of LVEF [12]. De Frutos showed that life-threatening arrhythmias did not correlate with the fibrosis detected by CMR, which was very rare in this subset of DCM pathogenic mutation, as previously observed.

Consistent with these findings, the latest ESC guidelines on Cardiomyopathies consider RBM20 genetic variants as a high arrhythmic genotype in DCM, with an annual SCD rate of 3–5%, suggesting ICD implantation with a higher threshold of common 35% [30].

Predictors of SCD are considered LVEF < 45% and LGE on CMR, which is a common marker of electrical instability in different heart diseases, but it is an infrequent finding in *RBM20* cardiomyopathy.

The severe manifestation of the disease was evident even in advanced HF outcomes, with a significant proportion of patients requiring heart transplantation. While both genders demonstrated comparable proband status and ICD implantation rates, males exhibited an earlier age of diagnosis and a greater degree of LV dysfunction, and in the studies specifying the gender of transplant recipients, approximately 96% were male. However, no significant disparities were observed in arrhythmic outcomes between males and females, emphasizing the need for further investigation into the underlying mechanisms driving sex-related differences in *RBM20* cardiomyopathy.

### 4.5. Limitation of the Study

The 3% prevalence of the *RBM20* cardiomyopathy in the DCM cohort is limited to only two studies included in the review that explored this aspect and did not account for contributions from smaller case series or case reports in the literature. However, more recent analyses of prevalence in familial disease, including 15 studies, suggest a slightly higher prevalence (~4%), with a possible overestimation due to limited verification of *RBM20* segregation ([31]).

## 5. Conclusions

*RBM20* cardiomyopathy is characterized by LV dysfunction and a high degree of electrical instability. Future research efforts will aim at elucidating the molecular mechanisms underlying *RBM20* variants, and their implications for disease pathogenesis are warranted. This will facilitate personalized management strategies and improve clinical outcomes in affected individuals.

## Figures and Tables

**Table 1:** Studies included in the systematic review.

Reference	Aim and Design of the Study	Study Population	Main Results	Pts with RBM20 Variant	Conclusions
Brauch KM, J Am Coll Cardiol. 2009 [5]	Authors conducted a genetic linkage analysis in two large families with autosomal dominant DCM to map a disease locus, eventually leading to the discovery of a variant hotspot in RNA-binding protein.	280 unrelated probands with DCM	*RBM20* variant carriers exhibit an earlier onset of disease compared to other familial DCM cases. *RBM20* cardiomyopathy is characterized by a high arrhythmic burden and the development of end-stage HF.	39 patients	*RBM20* accounted for 3% of DCM genetic variants and 13% of cases with history of SCD.
Refaat MM, Heart Rhythm. 2012 Mar [7]	To determine the prevalence of *RBM20* variants in a large, multiracial cohort of DCM pts. To analyse the clinical features and outcomes of variant carriers.	A total of 1465 subjects belonging to the GRADE database, including 283 patients with DCM	The prevalence of *RBM20* variants was 2.7% in individuals of European ancestry and 3.9% in individuals of African American ancestry. *RBM20* variant carriers have an increased risk of atrial fibrillation. Variant carriers had similar survival rates, HTx rates and frequencies of ICD therapy compared to non-variant carriers.”	8 patients	In this cohort of DCM patients, 3% of patients had an RBM20 variant. *RBM20* variants were not associated with adverse survival in terms of ventricular arrhythmias.
Van den Hoogenhof, Circulation. 2018 [8]	To investigate the underlying mechanism of arrhythmias in *RBM20* variant carriers.	18 *RBM20* variant carriers 22 *TTN* variant carriers	No differences between the two groups regarding ECG parameters and LV chamber dimension and function. Despite exhibiting similar levels of LV remodelling, *RBM20* variant carriers experienced a higher incidence of major ventricular arrhythmias. There was a significant presence of SCD family history in the *RBM20* variant carriers group.	18 patients	*RBM20* variant carriers have an increased risk of major ventricular arrhythmias compared to *TTN* variant carriers, suggesting that dysregulation of *TTN* constitutes not the sole mechanism in *RBM20* cardiomyopathy.
Hey TM, Circ Heart Fail. 2019 [9]	To further explore the phenotypic expression of patients carrying pathogenetic variants in the *RBM20* gene.	80 individuals carrying pathogenic *RBM20* variants across 15 families 30 familiar DCM patients with unknown genetic cause	The penetrance of *RBM20* cardiomyopathy was 66%. Asymptomatic family members were diagnosed with DCM at a younger age than the probands. A total of 30% of *RBM20* variant carriers experienced a life-threatening event. Male patients exhibited a more severe disease expression, but no differences were observed in arrhythmic outcomes. *RBM20* male patients had a shorter event-free survival compared to males with DCM of unknown genetic causes.	80 patients	The disease expression is severe, particularly in males, with earlier onset and ventricular dysfunction. The penetrance of the disease is also severe in family members, necessitating careful follow-up.
Parikh VN, Circ Heart Fail. 2019 [10]	To characterize clinical features of *RBM20* variant carriers.	Patients carrying different disease gene variants were analysed: A total of 74 patients with *RBM20* variant from an international registry (43 index cases) 633 patients affected with DCM 83 patients with *TTN* cardiomyopathy 87 patients with lamin (*LMNA*) cardiomyopathy	RBM20 index patients had a higher incidence of DCM and SCD compared to the general DCM cohort and the Titin (TTN) cohort. The rate of ventricular arrhythmias did not differ compared to patients with LMNA cardiomyopathy.	74 patients	Cardiomyopathy associated with *RBM20* has a severe phenotypic expression with early onset and high arrhythmic burden, sharing features similar to *LMNA* cardiomyopathy.
Robles-Mezcua A, Eur J Med Genet. 2021 [11]	To investigate the genotype-phenotype correlation in patients with *RBM20* gene variants.	8 patients carrying *RBM20* genetic variants	The main phenotype observed was DCM, except for one patient who was diagnosed with HCM. Overall, 62.5% had a history of cardiomyopathy among first-degree relatives, and 37.5% had a family history of SCD. No significant decline in systolic function was observed during the follow-up.	8 patients	*RBM20* cardiomyopathy is a highly penetrant disease with early onset and a frequent family history of DCM and SCD.
Cannie DE, Circ Genom Precis Med. 2023 [12]	To detail the natural history of *RBM20* gene carriers in comparison with a group of gene-elusive patients showing LV systolic dysfunction.	149 patients carrying a *RBM20* variant, including 32 probands 238 patients with gene-elusive systolic dysfunction	Men exhibited an earlier onset of cardiac disease with a greater degree of ventricular dilation and dysfunction compared to women. Among men and women with the *RBM20* variants, there was no difference in arrhythmic outcomes, while men more frequently experienced HF. In comparison to patients with variant-elusive systolic dysfunction, *RBM20* variant carriers had a 6-fold increase in the risk of ventricular arrhythmias and HF.	149 patients	*RBM20* cardiomyopathy exhibits high penetrance, even among relatives. Men display more severe phenotypes and a poorer prognosis, yet the arrhythmic burden is similar in both sexes. In *RBM20* variant carriers, ICD implantation should be considered for LVEF ≤45%.
De Frutos F, Eur Heart J Cardiovasc Imaging. 2023 [13]	To describe late LGE patterns according to genotypes in a cohort of DCM patients and to analyze the risk of major ventricular arrhythmias based on these patterns.	577 patients affected with DCM: 219 with P/LP variants; 358 gene elusive pts	LGE was absent or very rare in *RBM20* variant carriers. There was no significant association between the presence of LGE and lower LVEF. *RBM20* variant carriers experienced MVA even in absence of LGE.	22 patients	LGE is rare in patients with *RBM20* cardiomyopathy, and it does not correlate with the degree of electrical instability.

**Table 2:** Genetic variants reported in *n* = 8 studies included. B: Benign; LB: Likely Benign; VUS: Variant of Uncertain Significance; LP: Likely Pathogenic; P: Pathogenic. ACMG: American College of Medical Genetics and Genomics classification [14,15], NA = Not available.

Reference	HGVSc	HGVSp	Exon	ACMG	gnomAD
Brauch KM, J Am Coll Cardiol. 2009 [5]	c.1913C > T	p.(Pro638Leu)	9	P	0.00000354
	c.1901G > A	p.(Arg634Gln)	9	P	0.00000712
	c.1906C > A	p.(Arg636Ser)	9	P	NA
	c.1907G > A	p.(Arg634Gln)	9	P	0.00000712
	c.1909A > G	p.(Ser637Gly)	9	P	NA
Refaat MM, Heart Rhythm. 2012 Mar [7]	c.247C > A	p.(Leu83Ile)	2	LB	0.00000645
	c.1364C > T	p.(Ser455Leu)	4	B	0.0056
	c.1913C > T	p.(Arg636Ser)	9	P	NA
	c.2109G > C	p.(Arg703Ser)	9	VUS	NA
	c.2662G > A	p.(Asp888Asn)	11	B	0.00387
	c.3091G > T	p.(Gly1031Ter)	11	LP	NA
	c.3242C > G	p.(Pro1081Arg)	11	LB	NA
	c.3616G > A	p.(Glu1206Lys)	14	B	0.0000467
van den Hoogenhof, Circulation. 2018 [8]	c.769A > G	p.(Thr257Ala)	2	LB	NA
	c.1175G > A	p.(Arg392Gln)	2	LB	0.0000129
	c.1494C > A	p.(Ser498Arg)	5	LB	0.000013
	c.1760T > A	p.(Leu587His)	7	VUS	NA
	c.1900C > T	p.(Arg634Trp)	9	P	NA
	c.1913C > T	p.(Pro638Leu)	9	P	NA
	c.2042A > G	p.(Tyr681Cys)	9	B	0.000135
	c.3115C > T	p.(Pro1039Ser)	11	B	0.00021
Hey TM, Circ Heart Fail. 2019 [9]	c.1901G > A	p.(Arg634Gln)	9	P	0.00000712
	c.1907G > A	p.(Arg634Gln)	9	P	0.00000712
	c.1906C > A	p.(Arg636Ser)	9	P	NA
	c.1913C > T	p.(Arg636Ser)	9	P	NA
	c.2737G > A	p.(Glu913Lys)	11	P	NA
	c.586A > G	p.(Met196Val)	2	LB	0
	c.1174C > T	p.(Arg392Trp)	2	VUS	0.0000129
	c.2021A > G	p.(Asp674Gly)	9	LB	0.00000644
	c.3115C > T	p.(Pro1039Ser)	11	B	0.00021
Parikh VN, Circ Heart Fail. 2019 [10]	c.2721–2760 region	p.907–920 region	11	NA	NA
	c.1881–1920 region	p.627–640 region	9	NA	NA
Robles-Mezcua A, Eur J Med Genet. 2021 [11]	c.1793A > C	p.(Gln598Pro)	7	VUS	NA
	c.776G > T	p.(Gly259Val)	2	LB	0.000013
	c.3169C > T	p.(Arg1057Trp)	11	B	0.000102
	c.580A > G	p.(Met194Val)	2	LB	NA
	c.529A > T	p.(Thr177Ser)	2	B	0.0000896
	c.154C > A	p.(Pro52Thr)	1	LB	NA
Cannie DE, Circ Genom Precis Med. 2023 [12]	c.1906C > A	p.(Arg636Ser)	9	P	NA
	c.1907G > A	p.(Arg634Gln)	9	P	0.00000712
	c.1901G > A	p.(Arg634Gln)	9	P	0.00000712
	c.2737G > A	p.(Glu913Lys)	11	P	NA
	c.1913C > T	p.(Arg636Ser)	9	P	NA
	c.1900C > T	p.(Arg634Trp)	9	P	NA
	c.2746G > A	p.(Glu916Lys)	11	VUS	NA
	c.2723T > C	p.(Leu908Pro)	11	VUS	NA
De Frutos F, Eur Heart J Cardiovasc Imaging. 2023 [13]	NA	NA	NA	NA	NA

## References

[1] McDonagh T.A., Metra M., Adamo M., Gardner R.S., Baumbach A., Böhm M., Burri H., Butler J., Čelutkienė J., Chioncel O. (2021). 2021 ESC Guidelines for the Diagnosis and Treatment of Acute and Chronic Heart Failure. Eur. Heart J..

[2] Burkett E.L., Hershberger R.E. (2005). Clinical and Genetic Issues in Familial Dilated Cardiomyopathy. J. Am. Coll. Cardiol..

[3] Akinrinade O., Ollila L., Vattulainen S., Tallila J., Gentile M., Salmenperä P., Koillinen H., Kaartinen M., Nieminen M.S., Myllykangas S. (2015). Genetics and Genotype-Phenotype Correlations in Finnish Patients with Dilated Cardiomyopathy. Eur. Heart J..

[4] Hershberger R.E., Hedges D.J., Morales A. (2013). Dilated Cardiomyopathy: The Complexity of a Diverse Genetic Architecture. Nat. Rev. Cardiol..

[5] Brauch K.M., Karst M.L., Herron K.J., de Andrade M., Pellikka P.A., Rodeheffer R.J., Michels V.V., Olson T.M. (2009). Mutations in Ribonucleic Acid Binding Protein Gene Cause Familial Dilated Cardiomyopathy. J. Am. Coll. Cardiol..

[6] Wyles S.P., Li X., Hrstka S.C., Reyes S., Oommen S., Beraldi R., Edwards J., Terzic A., Olson T.M., Nelson T.J. (2016). Modeling Structural and Functional Deficiencies of RBM20 Familial Dilated Cardiomyopathy Using Human Induced Pluripotent Stem Cells. Hum. Mol. Genet..

[7] Refaat M.M., Lubitz S.A., Makino S., Islam Z., Frangiskakis J.M., Mehdi H., Gutmann R., Zhang M.L., Bloom H.L., MacRae C.A. (2012). Genetic Variation in the Alternative Splicing Regulator RBM20 Is Associated with Dilated Cardiomyopathy. Heart Rhythm..

[8] van den Hoogenhof M.M.G., Beqqali A., Amin A.S., van der Made I., Aufiero S., Khan M.A.F., Schumacher C.A., Jansweijer J.A., van Spaendonck-Zwarts K.Y., Remme C.A. (2018). RBM20 Mutations Induce an Arrhythmogenic Dilated Cardiomyopathy Related to Disturbed Calcium Handling. Circulation.

[9] Hey T.M., Rasmussen T.B., Madsen T., Aagaard M.M., Harbo M., Mølgaard H., Møller J.E., Eiskjær H., Mogensen J. (2019). Pathogenic RBM20-Variants Are Associated With a Severe Disease Expression in Male Patients With Dilated Cardiomyopathy. Circ. Heart Fail..

[10] Parikh V.N., Caleshu C., Reuter C., Lazzeroni L.C., Ingles J., Garcia J., McCaleb K., Adesiyun T., Sedaghat-Hamedani F., Kumar S. (2019). Regional Variation in RBM20 Causes a Highly Penetrant Arrhythmogenic Cardiomyopathy. Circ. Heart Fail..

[11] Robles-Mezcua A., Rodríguez-Miranda L., Morcillo-Hidalgo L., Jiménez-Navarro M., García-Pinilla J.M. (2021). Phenotype and Progression among Patients with Dilated Cardiomyopathy and RBM20 Mutations. Eur. J. Med. Genet..

[12] Cannie D.E., Protonotarios A., Bakalakos A., Syrris P., Lorenzini M., De Stavola B., Bjerregaard L., Dybro A.M., Hey T.M., Hansen F.G. (2023). Risks of Ventricular Arrhythmia and Heart Failure in Carriers of RBM20 Variants. Circ. Genom. Precis. Med..

[13] de Frutos F., Ochoa J.P., Fernández A.I., Gallego-Delgado M., Navarro-Peñalver M., Casas G., Basurte M.T., Larrañaga-Moreira J.M., Mogollón M.V., Robles-Mezcua A. (2023). Late Gadolinium Enhancement Distribution Patterns in Non-Ischaemic Dilated Cardiomyopathy: Genotype-Phenotype Correlation. Eur. Heart J. Cardiovasc. Imaging.

[14] Hershberger R.E., Givertz M.M., Ho C.Y., Judge D.P., Kantor P.F., McBride K.L., Morales A., Taylor M.R.G., Vatta M., Ware S.M. (2018). Genetic evaluation of cardiomyopathy: A clinical practice resource of the American College of Medical Genetics and Genomics (ACMG). Genet. Med..

[15] Richards S., Aziz N., Bale S., Bick D., Das S., Gastier-Foster J., Grody W.W., Hegde M., Lyon E., Spector E. (2015). Standards and guidelines for the interpretation of sequence variants: A joint consensus recommendation of the American College of Medical Genetics and Genomics and the Association for Molecular Pathology. Genet. Med..

[16] Schultheiss H.-P., Fairweather D., Caforio A.L.P., Escher F., Hershberger R.E., Lipshultz S.E., Liu P.P., Matsumori A., Mazzanti A., McMurray J. (2019). Dilated Cardiomyopathy. Nat. Rev. Dis. Primers.

[17] Wang Y., Dobreva G. (2023). Epigenetics in LMNA-Related Cardiomyopathy. Cells.

[18] Li S., Guo W., Dewey C.N., Greaser M.L. (2013). Rbm20 Regulates Titin Alternative Splicing as a Splicing Repressor. Nucleic Acids Res..

[19] Guo W., Schafer S., Greaser M.L., Radke M.H., Liss M., Govindarajan T., Maatz H., Schulz H., Li S., Parrish A.M. (2012). RBM20, a Gene for Hereditary Cardiomyopathy, Regulates Titin Splicing. Nat. Med..

[20] Mutations of the Cardiac Ryanodine Receptor (RyR2) Gene in Familial Polymorphic Ventricular Tachycardia—PubMed. https://pubmed.ncbi.nlm.nih.gov/11157710/.

[21] Zhang M., Gao H., Liu D., Zhong X., Shi X., Yu P., Jin L., Liu Y., Tang Y., Song Y. (2019). CaMKII-Δ9 Promotes Cardiomyopathy through Disrupting UBE2T-Dependent DNA Repair. Nat. Cell Biol..

[22] Murayama R., Kimura-Asami M., Togo-Ohno M., Yamasaki-Kato Y., Naruse T.K., Yamamoto T., Hayashi T., Ai T., Spoonamore K.G., Kovacs R.J. (2018). Phosphorylation of the RSRSP Stretch Is Critical for Splicing Regulation by RNA-Binding Motif Protein 20 (RBM20) through Nuclear Localization. Sci. Rep..

[23] Ihara K., Sasano T., Hiraoka Y., Togo-Ohno M., Soejima Y., Sawabe M., Tsuchiya M., Ogawa H., Furukawa T., Kuroyanagi H. (2020). A Missense Mutation in the RSRSP Stretch of Rbm20 Causes Dilated Cardiomyopathy and Atrial Fibrillation in Mice. Sci. Rep..

[24] Gaertner A., Klauke B., Felski E., Kassner A., Brodehl A., Gerdes D., Stanasiuk C., Ebbinghaus H., Schulz U., Dubowy K.-O. (2020). Cardiomyopathy-Associated Mutations in the RS Domain Affect Nuclear Localization of RBM20. Hum. Mutat..

[25] Schneider J.W., Oommen S., Qureshi M.Y., Goetsch S.C., Pease D.R., Sundsbak R.S., Guo W., Sun M., Sun H., Kuroyanagi H. (2020). Dysregulated Ribonucleoprotein Granules Promote Cardiomyopathy in RBM20 Gene-Edited Pigs. Nat. Med..

[26] Pantou M.P., Gourzi P., Gkouziouta A., Tsiapras D., Zygouri C., Constantoulakis P., Adamopoulos S., Degiannis D. (2018). Phenotypic Heterogeneity within Members of a Family Carrying the Same RBM20 Mutation R634W. Cardiology.

[27] Dec G.W., Fuster V. (1994). Idiopathic Dilated Cardiomyopathy. N. Engl. J. Med..

[28] Ollila L., Nikus K., Holmström M., Jalanko M., Jurkko R., Kaartinen M., Koskenvuo J., Kuusisto J., Kärkkäinen S., Palojoki E. (2017). Clinical Disease Presentation and ECG Characteristics of LMNA Mutation Carriers. Open Heart.

[29] Cipriani A., Bauce B., De Lazzari M., Rigato I., Bariani R., Meneghin S., Pilichou K., Motta R., Aliberti C., Thiene G. (2020). Arrhythmogenic Right Ventricular Cardiomyopathy: Characterization of Left Ventricular Phenotype and Differential Diagnosis With Dilated Cardiomyopathy. J. Am. Heart Assoc..

[30] Arbelo E., Protonotarios A., Gimeno J.R., Arbustini E., Barriales-Villa R., Basso C., Bezzina C.R., Biagini E., Blom N.A., de Boer R.A. (2023). 2023 ESC Guidelines for the Management of Cardiomyopathies. Eur. Heart J..

[31] Gregorich Z.R., Zhang Y., Kamp T.J., Granzier H.L., Guo W. (2024). Mechanisms of RBM20 Cardiomyopathy: Insights From Model Systems. Circ. Genom. Precis. Med..

